# International Perception of Competence, Education, and Training Needs Among Biomedical Professionals Involved in Medicines Development

**DOI:** 10.3389/fphar.2019.00188

**Published:** 2019-03-05

**Authors:** Kyoko Imamura, Domenico Criscuolo, Anna Jurczynska, Gustavo Kesselring, Peter Stonier, Tatsushi Tsuda, Honorio Silva

**Affiliations:** ^1^Social Cooperation Program of IT Healthcare, Graduate School of Pharmaceutical Sciences, The University of Tokyo, Tokyo, Japan; ^2^Italian Association of Pharmaceutical Medicine (SIMeF), Milan, Italy; ^3^Spanish Association of Pharmaceutical Medicine (AMIFE), Madrid, Spain; ^4^Faculty of Life Sciences & Medicine, King’s College London, London, United Kingdom; ^5^Faculty of Pharmaceutical Medicine, London, United Kingdom; ^6^IFAPP Academy, New York, NY, United States

**Keywords:** competence, education, training, pharmaceutical physicians, medicines development, IFAPP, pharmatrain, pharmaceutical medicine

## Abstract

The development of new medicines today, requires a multi-professional workforce, both in industry and the clinical research arena. Pharmaceutical physicians (PPs) and medicines development scientists (MDS) need a certain level of competence, achieved through on-the-job experience, with a postgraduate education foundation and continuous professional development programs. In order to assess the self-perception of competence, education and training needs, an on-line questionnaire based on the seven domains of competence, developed by IFAPP-PharmaTrain, was prepared and distributed among PPs and MDS members of IFAPP’s affiliated professional associations in countries with facilities for postgraduate education. The data collection was run over a fixed period of three months in Japan, Italy, Brazil, and Spain during 2017. Results indicate low but variable levels of perceived competence for the various domains as well as seniority in the job. All respondents declared a significant need for continuing professional development in all domains. These results corroborate and support the continuous efforts, put in place by IFAPP and the PharmaTrain Federation, to foster the development of accredited education and training among professionals involved in medicines development.

## Introduction

For some time now, the biopharmaceutical industry has been the key link between basic biomedical discovery and the emergence of novel medicines that prolong or improve life. However, the industry faces several ongoing and emerging challenges, including technical knowledge gaps, limitations in clinical testing, lowered productivity, higher development costs, increased regulatory requirements, growing payer pressures and patent expiration.

The lack of an adequately sized and appropriately trained multi-professional workforce, both in the industry and the clinical research field, is also a significant part of the problem. There is a perceived mismatch between the profiles and abilities of graduates from academic programs in healthcare professions, and the changing needs of the various health systems around the world. As a possible solution to achieving a transformative learning, an outcomes-based education, or competency-based education (CBE), has been proposed (Silva et al., 2013). Competent professionals would be able to perform their specific responsibilities effectively, such as bringing and maintaining new medicines to the marketplace. A need for competency-based education and training has been identified in the United States, Europe, and Latin America (Dubois et al., 2016).

These respective professional groups have been left with the responsibility to define the competencies needed to perform their function effectively. Competencies can be clustered in domains and can be learned through proper postgraduate education or continuing professional development (CPD) (Sonstein et al., 2014).

The International Federation of Associations of Pharmaceutical Physicians and Pharmaceutical Medicine (IFAPP)^1^ and the PharmaTrain Federation (PharmaTrain)^2^ assumed the task of producing the defined core competencies to orientate Pharmaceutical Medicine and Medicines Development as a discipline and profession. Three areas, seven domains and 57 core competencies at the cognitive level, were identified (Silva et al., 2013). The domains have been summarized in a Statement of Competence.

In addition to serving as a template for job profiles and portfolios, the domains can be used to identify general education and training needs. Based on these premises, an international survey among members of the IFAPP national member association was designed using an online questionnaire. Stakeholders were asked about their self-perception of competence and the need for education and training. The results were then assessed to identify gaps, in order to address the potential need for future development of pharmaceutical physicians and medicines development scientists.

## Materials and Methods

### Development of On-Line Questionnaire

The questionnaire was developed based on the previously defined domains for competence in medicines development (Silva et al., 2013). An invitation to participate in the survey was sent to a defined list of members and non-members of the pharmaceutical medicine national associations in Brazil, Japan, Spain, and Italy. The responders who agreed to participate in this survey were asked for their demographic data (functional area, place of employment, level of experience, association with IFAPP’s national member association) and their self-assessment of each of the seven domains for core professional competence. For each domain, the responders were asked their competence level (level-1 as “fundamental awareness” (basic awareness) to level-5 as “expert”), and its significance to their position (from “very low” to “very high”), in a Likert Scale as well as their training needs (Yes/No). The survey was conducted in an anonymous manner.

Responders were provided with a statement of competence defined by IFAPP, as well as a short description of each domain, to help their understanding of these as referred to in the survey. All questions and multiple-choice answers were developed using the Google on-line questionnaire format.

Definition of the domains (IFAPP-PharmaTrain Federation Collaboration Working Group, 2016) was used as per the following statement of competence.

### Domain 1: Discovery of Medicines and Early Development

The Pharmaceutical Physician / Medicines Development Scientist can identify unmet therapeutic needs, evaluate the evidence for a new candidate for clinical development and design a Clinical Development Plan for a Target Product Profile.

### Domain 2: Clinical Development and Clinical Trials

The Pharmaceutical Physician / Medicines Development Scientist can design, execute and evaluate exploratory and confirmatory clinical trials and prepare manuscripts or reports for publication and regulatory submissions.

### Domain 3: Medicines Regulation

The Pharmaceutical Physician / Medicines Development Scientist can interpret effectively the regulatory requirements for the clinical development of a new drug through the product life-cycle to ensure its appropriate therapeutic use and proper risk management.

### Domain 4: Drug Safety Surveillance

The Pharmaceutical Physician / Medicines Development Scientist can evaluate the choice, application and analysis of post-authorization surveillance methods to meet the requirements of national/international agencies for proper information and risk minimization to patients and clinical trial subjects.

### Domain 5: Ethics and Subject Protection

The Pharmaceutical Physician / Medicines Development Scientist can combine the principles of clinical research and business ethics for the conduct of clinical trials and commercial operations within the organization.

### Domain 6: Healthcare Marketplace

The Pharmaceutical Physician / Medicines Development Scientist can appraise the pharmaceutical business activities in the healthcare environment to ensure that they remain appropriate, ethical and legal to keep the welfare of patients and subjects at the forefront of decision making in the promotion of medicines and design of clinical trials.

### Domain 7: Communication and Management

The Pharmaceutical Physician / Medicines Development Scientist can interpret the principles and practices of people management and leadership, using effective communication techniques and interpersonal skills to influence key stakeholders and achieve the scientific and business objectives.

### Target Population

To maximize the participation from expected stakeholders in medicines development, a standard letter to explain the objectives of this survey was distributed by the IFAPP’s national member associations in the above-mentioned countries. In addition, the questionnaire was posted on the IFAPP website to encourage individual participation from other member associations as well as for those possible responders not affiliated to IFAPP national member associations.

### Study Period

Each national member association posted the questionnaire for three months. The questionnaire was first posted on-line in Japan (started on February 27th and terminated on May 31st, 2017), followed by Italy, Spain, and Brazil. During the posted period, representatives of the national member associations sent out reminder e-mails to their members to encourage participation. By the end of November 2017, the entire survey was completed, and the responses were analyzed.

### Statistical Analysis Methods

The results for the perception of “competency level” included combined responses of 1, 2, and 3 from the competence level as a composite score of “0” (i.e., “less than competent”), and translated combined responses of 4 or 5 into a composite score of “1” (i.e., “competent”). This scale was also used for perceived “significance to one’s position.” For the questions regarding the “training need” per domain, “1” indicated “yes” and “0” indicated “no.”

## Results

### Responses for Analysis

In total, 680 full responses were obtained in this global survey. The number of responses were 388 in Japan, 194 in Italy, 61 in Spain, and 34 in Brazil. From the open survey posted on the IFAPP website, individual responses were sent from Korea, Philippines and Greece (one response each).

### Demographics of the Respondents

Overall demographics of the respondents are shown in Supplementary Table S1.

#### Overall Statistics

The 46% of the respondents were working in clinical research, followed by medical affairs (11%). When classified by the place of employment, 54% of the respondents were working in sponsor organizations (pharmaceutical company / biotech company), followed by contract research organizations (CROs- 26%). In terms of their level of experience, the majority had over 10 years of experience except in Japan, half of whom had less than 10 years of experience. Most Japanese respondents were from clinical research, whereas more medical affairs respondents were from Italy.

#### Functional Areas and Level of Experience

**FIGURE 1:**
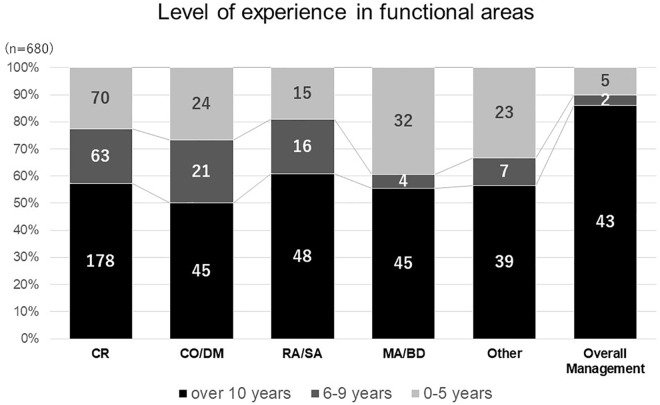
Level of experience in functional areas. clinical research (CR), clinical operation and data management (CO DM), regulatory affairs and safety affairs (RA SA), medical affairs and business development (MABD).

As the number of responses obtained from nine different functional areas were not evenly distributed, respondents were divided into six main categories according to their functional areas: clinical research, clinical operations and data management, regulatory affairs and safety, medical affairs and business development, overall management and others in consideration of the similarity in knowledge, skill and attitudes required in their functional areas. Less experienced respondents (less than 10 years) were found in the medical affairs and business development areas (Figure 1). On the contrary, most respondents involved in overall management had over 10 years of experience. With regards to national differences, most respondents from clinical research and medical affairs had over 10 years’ experience across all countries.

#### Place of Employment and Level of Experience

Most respondents in sponsor organizations (69%) had over 10 years of experience, compared the experience of those working for CROs (56% reported less than 10 years). The proportion of respondents in management positions was somewhat similar: 9% in sponsor organization and 6% in CROs.

### Overall Results of Competency Level, Significance to One’s Position, and Training Needs Related to Domains

**FIGURE 2:**
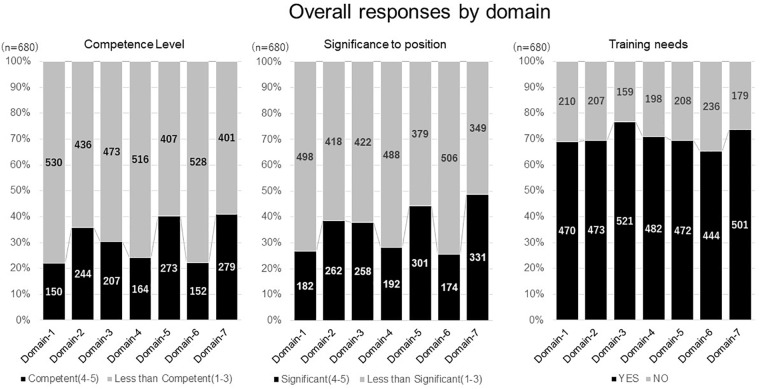
Overall responses by domain. Domain-1: Discovery of Medicines and Early Development, Domain-2: Clinical Development and Clinical Trials, Domain-3: Medicines Regulation, Domain-4: Drug Safety Surveillance, Domain-5: Ethics and Subject Protection, Domain-6: Healthcare Marketplace, Domain-7: Communication and Management.

Survey responses concerning competence level, significance to position, and training needs are shown in Figure 2. The perception of competence varied among domains, though did not exceed 50% for any of them. A similar response, related to relevance to the job, was also found. Domains 2, 5, and 7 shared relatively higher rates in competence level and significance to their position, suggesting a close relevance between these two perceptions. In contrast to approximately 30% of respondents who felt less competent, and the significance to their position, high training needs (nearly 70%) was reported in all domains. In relation to the number of years of experience, less experienced respondents perceived higher training needs.

When compared across the seven domains of competencies, a higher perception of competence level and significance to their position were observed in functional areas related to medical affairs and business development, followed by regulatory affairs and safety. In clinical development related areas (clinical research, clinical operation, and data management), a perception of competence and significance to their position, were relatively lower in matching domains 1 and 2.

## Discussion

The need and value of competency-based education and training has become internationally recognized in a variety of fields, from an economic viewpoint and a broader social perspective (OECD, 2005). In the pharmaceutical industry, cognitive education has been structured and delivered in accordance with the standardized syllabus and core curriculum and promoted by the IFAPP and PharmaTrain. On-line educational programs in pharmaceutical medicine and medical affairs has also been developed by the IFAPP Academy^3^, created as the educational arm of IFAPP, as a strategic collaboration with King’s College London^4^. Recently, the IFAPP and PharmaTrain defined core competencies (Silva et al., 2013; IFAPP-PharmaTrain Federation Collaboration Working Group, 2016), according to which this survey was conducted, to seek stakeholders’ view of the current status of competency-based education in this profession.

With regards to study limitations, which may have affected the generalizability of our observations, we missed responses from major countries active in medicines development such as the United States and United Kingdom. Secondly, the majority of responses came from highly experienced professionals in clinical development related functional areas, employed by sponsor organizations and CROs, which made a comparative analysis difficult. Thirdly, since the study was designed based on a non-probabilistic sample, as one single observation with different sizes for the national cohorts, analysis of the results requires caution. For example, more than half of the responses recorded came from Japan, where the culture of professional development and hiring opportunities differ from the other three countries. As a result, the overall respondents in medical affairs and business development appeared relatively less experienced due to proportionately more Japanese respondents (Figure 1), where the hiring of pharmaceutical physicians and medicines development scientists is a rather new area and possibly attracts relatively less experienced persons. Last but not least, the overall perception of competence was generally lower than 50% across the domains and precluded further analysis.

In terms of the common observations obtained within the limits, the overall analysis showed that the level of perceived competence in clinical development related domains (1 and 2) was lower in those working in CROs, compared to those working in sponsor organizations. As clinical development tasks are increasingly outsourced to CROs, their training should be considered in order to improve overall performance in medicines development. A high interest in training was also observed in all four participating countries across the domains, despite the relatively longer years of working experiences of the respondents, suggesting that this could be considered a global need. Industrial restructuring could be partially attributable to the loss of resources from workplaces, such as experienced mentors and the educational budget, as well as a changing environment for medicines development which requires new competencies in diverse areas. It should be noted that a similar study of a larger sample, contributed to by clinical research professionals from all over the world showed comparable findings, with significant variations among the respondents’ perceived competence and relevance of domains and competencies as well as training needs for the various professional roles involved in clinical trials (Sonstein et al., 2016).

The results are indicative of the need for a more thorough confirmation on a country-by-country basis and a call for attention to all stakeholders. To promote competency-based education and training in a real-world setting, development of standardized assessment tools may add value. As the concept of Entrustable Professional Activities (EPA) has been adopted in a variety of areas of professional education, notably in medicine (Cate, 2013) and pharmacy (Pittenger et al., 2016), having a common currency for training may help to create a common understanding among stakeholders as well as mutual recognition of training offered by a variety of providers, as is proposed for residency training (Englander et al., 2014).

In summary, missing areas and opportunities for education and training can be identified in national surveys using the common definition of competencies and compared based on the understanding of the differences in cultural backgrounds and job markets. Opportunities for improvement could be provided with a standardized assessment in order to meet the expected level of competence for professionals in pharmaceutical medicine and medicines development.

## Data Availability

The raw data supporting the conclusions of this manuscript will be made available by the authors, without undue reservation, to any qualified researcher.

## Author Contributions

KI, TT, and HS contributed substantial to conceived and designed the work, analyzed and interpreted the data. KI, HS, DC, GK, and AJ collected the data and the reviewed the manuscript. PS and HS revised the work critically for important intellectual content. All authors provided final approval of the work.

## Conflict of Interest Statement

The authors declare that the research was conducted in the absence of any commercial or financial relationships that could be construed as a potential conflict of interest.

## Footnotes

1 http://ifapp.org/

2 https://www.pharmatrain.eu/

3 https://ifappacademy.org/

4 https://www.kcl.ac.uk/index.aspx
