# Supplementary material for: International Perception of Competence, Education, and Training Needs Among Biomedical Professionals Involved in Medicines Development
Source: Front Pharmacol. 2019 Mar 5;10:188. doi: 10.3389/fphar.2019.00188 (PMC6411843; doi:10.3389/fphar.2019.00188)
Supplement: Supplementary file 1 [file Table_1.pdf]

Table S1: Demographics of respondents

Demographics	Total (n=680)	
Functional Area (Key area of work)		
1) Clinical Research	311	46%
2) Medical Affairs	76	11%
3) Regulatory Affairs	29	4%
4) Business Development	5	1%
5) Safety and Risk Management (Safety Affairs)	50	7%
6) Data Management	38	6%
7) Clinical Operations	52	8%
8) Overall Management	50	7%
9) Other	69	10%
Place of Employment		
1) Sponsor (Pharma / Biotech) Organization	364	54%
2) Contract Research Organization (CRO)	177	26%
3) Investigational Site	16	2%
4) Academic Medical Center	33	5%
5) Academic Institution	15	2%
6) Public Hospital	11	2%
7) Private Hospital	1	0%
8) Regulatory Agency	1	0%
9) Professional Associations	5	1%
10) Other	57	8%
Level of Experience		
1) 0-2 years	75	11%
2) 3-5 years	94	14%
3) 6-9 years	113	17%
4) over 10 years	398	59%
Member of IFAPP national member association*		
1) Yes	74	26%
2) No	216	74%

*: Not detected in Japan
